# The association between regional anesthesia and postoperative pulmonary complications following lung resection surgery: a hospital-based, retrospective cohort study

**DOI:** 10.1080/07853890.2026.2677995

**Published:** 2026-05-26

**Authors:** Jing-Jing Sun, Shao-Xian Liang, Lu Xu, Lin-Cong Zhao, Xue-Sheng Liu, Wan-Shui Yang, Yao Lu

**Affiliations:** ^a^ Department of Anesthesiology, The First Affiliated Hospital of Anhui Medical University, Hefei, China; ^b^ Department of Nutrition, School of Public Health, Anhui Medical University, Hefei, China; ^c^ Anhui Provincial Key Laboratory of Population Health and Aristogenics/Key Laboratory of Environmental Toxicology of Anhui Higher Education Institutes, Anhui Medical University, Hefei, China

**Keywords:** General anesthesia, regional anesthesia, lung resection surgery, postoperative pulmonary complications, major adverse cardiac events

## Abstract

**Background:**

Regional anesthesia (RA) are extensively utilized in lung-resection surgery for pain relief. However, the associations between RA and postoperative pulmonary complications (PPCs) remain inconclusive. Therefore, we aimed to investigate the associations between RA and the incidence of PPCs in adults undergoing lung-resection surgery.

**Methods:**

This retrospective cohort study included adult patients who underwent lung-resection surgery under general anesthesia (GA) at a large comprehensive teaching hospital in China. The primary outcome was PPCs within 7 days.

**Results:**

A total of 9,208 patients were enrolled and assigned into group GA, which received GA alone, or group GA+RA, which received GA combined with RA. Within the GA+RA group, patients were further categorized into the GA + PRA group (GA combined with peripheral regional anesthesia) and the GA + EA group (GA combined with epidural anesthesia). Following matching, the GA+RA group showed a non-significant trend toward lower incidence of PPCs (HR 0.93; 95% CI, 0.86–1.01; *p* = 0.078). However, a significant decreasing trend was observed in specific subgroups, including patients with a surgical duration ≤ 2 h (HR, 0.74 [0.64–0.86]) and those undergoing thoracoscopic surgery (HR, 0.90 [0.83–0.98]). GA combined with PRA also reduced the risk of postoperative congestive heart failure (HR, 0.37 [0.20–0.68]) and pain (HR, 0.89 [0.84–0.95]).

**Conclusions:**

In this retrospective cohort study, the addition of RA to GA was not associated with a significantreduction in PPCs within 7 days after lung resection compared to GA alone. Exploratory analyses identified potential subgroup-specific benefits that warrant prospective validation.

**Trial registration:**

Chinese Clinical Trial Registry (ChiCTR2400090895).

## Graphical abstract

**Figure:**
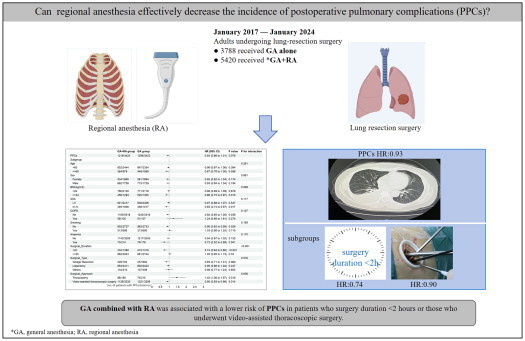

## Introduction

By 2022, approximately 4,820,000 new cancer cases and 3,210,000 cancer deaths had occurred in China [1]. Lung cancer is the most common type of cancer and the leading cause of cancer-related mortality in China [1]. Currently, the National Comprehensive Cancer Network (NCCN) guidelines recommend surgery as the standard treatment for early-stage lung cancer, particularly for stages IA, IIA, and IIIA [2]. Postoperative pulmonary complications (PPCs) are prevalent, occurring in 14–59% of patients who underwent thoracic surgery, with high morbidity. These complications are the primary causes of postoperative morbidity, postoperative mortality, and prolonged hospital stays [3,4].

Regional anesthetic (RA) techniques, such as epidural or peripheral nerve blocks, serve as cornerstones of multimodal analgesia, and these techniques are widely utilized in thoracic surgery [5]. For open thoracotomy, thoracic epidural analgesia (TEA) is strongly recommended as the gold-standard analgesic technique for enhanced recovery after lung surgery, according to the current clinical practice guidelines [6]—jointly published by the ERAS Society and the European Society of Thoracic Surgeons (ESTS). In contrast, regional blocks are preferred for video-assisted thoracoscopic surgery (VATS) or when TEA is not suitable, because of similar pain control and better hemodynamic stability. However, real-world evidence on the use of RA for preventing PPCs is inconsistent [7–9]. Some studies have suggested that RA reduces the incidence of PPCs and cardiac events [10,11]. In contrast, a multicenter study revealed higher unadjusted rates of PPC with RA (42.1% vs. 30.9%), which became nonsignificant after adjustment [12].

Therefore, in this study, we evaluated the association between general anesthesia combined with RA and the incidence of PPCs in patients who underwent lung resection surgery. To minimize the influence of confounding factors on PPCs, we incorporated relevant variables into our analysis. PPCs are influenced by multiple factors, including surgical and anesthetic techniques and preoperative patient conditions [13–15].

## Methods

### Study populations and inclusion criteria

This retrospective cohort study included adults aged 18 years or older who underwent lung resection surgery between January 8, 2017, and January 1, 2024, at the First Affiliated Hospital of Anhui Medical University, a large comprehensive teaching hospital in China. Their data were retrospectively collected and analyzed in October 2024.

The patients included in this study were all adults who underwent lung resection under general anesthesia. We excluded patients who were younger than 18 years at the time of surgery; who underwent combined abdominal, esophageal, or cardiac surgeries; who underwent emergency procedures; who underwent transplant surgeries; who received anesthesia other than general anesthesia; or who were classified as having a physical status of class 5 or 6 according to the methods of the American Society of Anesthesiologists (ASA) [16], indicating that the patients were moribund or brain-dead organ donors.

This study was approved by the Institutional Review Board of the First Affiliated Hospital of Anhui Medical University (Approval No. PJ2024-07-76), which waived the requirement for informed consent because of the retrospective nature of the research. This work is reported in accordance with the STROCSS criteria [17]. This study was conducted in adherence to the principles in the Declaration of Helsinki.

### Baseline characteristics

The characteristics included potential confounders such as demographic factors (age, sex, ASA, body mass index), comorbid conditions (hypertension, diabetes, smoking history, alcohol consumption history, chronic obstructive pulmonary disease (COPD), emphysema, pulmonary arterial hypertension, and pulmonary diseases such as pulmonary embolism and congenital pulmonary vascular malformation), preoperative medications (steroids), baseline laboratory data (hemoglobin and albumin levels), and intraoperative data (type of surgery, type of anesthesia, surgical approach, emergency status, duration of surgery and anesthesia, etc.).

### Outcomes

Postoperative pulmonary complications were identified if at least one relevant event was recorded in the HIS. Given the variability of the international diagnostic criteria for PPCs, comprehensive diagnostic criteria were formulated by combining the European standards with the clinical practice guidelines of our hospital. These comprehensive criteria included diagnoses such as pneumonia, atelectasis, respiratory failure, pleural effusion, pneumothorax, bronchospasm, and aspiration pneumonitis (Table S1) [18,19]. The secondary outcomes included major adverse cardiac events (MACEs). MACEs were defined as new-onset cardiac arrhythmia, acute myocardial infarction, congestive heart failure, angina, postoperative myocardial injury, and nonfatal cardiac arrest [18] (Table S2). These events also included common complications following thoracic surgery, such as acute kidney injury (AKI) [20], hypotension, bradycardia, and pain [21,22], as well as unplanned intubation, unexpected ICU admission, unplanned discharge, hospitalization cost, and postoperative hospital stay duration. All outcomes were assessed from the date of the procedure until 7 days postoperatively.

### Statistical analysis

Categorical variables are presented as frequencies and percentages. Continuous quantitative variables are reported as means ± standard deviations (SDs) (normally distributed data) or medians [interquartile ranges] (non-normally distributed data). Baseline characteristics were compared before and after propensity score matching. Covariate balance was evaluated using the absolute standardized mean difference (ASMD), with ASMD < 0.1 indicating acceptable matching (Table 1 and 2). Propensity scores were estimated using a multivariable logistic regression model, with the receipt of regional anesthesia (yes/no) as the dependent variable. Covariates were selected a priori on the basis of their clinical relevance as predictors of RA use or PPC risk (Figure S4). This approach aimed to minimize selection bias. Patients were matched 1:1 using a greedy algorithm without replacement, with a caliper width of 0.2 SD of the logit of the propensity score. For the primary and secondary outcomes, we applied the same doubly robust approach, namely propensity score matching, followed by further adjustment for the propensity score in the Cox model. Hazard ratios (HRs) were estimated using cause-specific Cox proportional hazards regression. To enhance the interpretability and clinical applicability of our findings, PRA techniques were classified into four subtypes: (1) paravertebral block (PVB), which involves the ultrasound-guided or landmark-based injection of the local anesthetic into the thoracic paravertebral space, providing ipsilateral somatic and sympathetic blockade; (2) serratus anterior plane block (SAPB), which involves the ultrasound-guided injection of the local anesthetic into the fascial plane superficial or deep to the serratus anterior muscle at the level of the fifth rib in the midaxillary line. SAPB blocks the lateral cutaneous branches of the T2–T9 intercostal nerves, providing hemithoracic analgesia; (3) intercostal nerve block (ICNB), which involves the ultrasound-guided injection of the local anesthetic at multiple levels along the intercostal space, providing segmental analgesia; and (4) other techniques, including erector spinae plane block, pectoral nerve block, or combinations of the aforementioned techniques. Owing to the limited sample sizes, these patients were analyzed as a combined group. Prespecified subgroup analyses were performed on the matched cohort with further propensity score adjustment in the Cox model; this cohort was stratified by age (18–65 and ≥65 years), sex (female and male), BMI (<24 and ≥24 kg/m^2^) [23], ASA stage (I–II and III–IV), presence of COPD (yes and no), smoking history (yes and no), presence of anemia (yes and no), surgical duration (<2 h and ≥2 h), surgical type (wedge resection, lobectomy, and others), and surgical approach (video-assisted thoracoscopic surgery and thoracotomy). The likelihood ratio test was used to investigate significant multiplicative interactions. Subgroup analyses were exploratory (not prespecified in the study protocol) and should be interpreted as hypothesis-generating. To assess the robustness of the results to misclassification bias, we performed a sensitivity analysis using a restrictive PPC definition, excluding cases classified solely as ‘suspected pulmonary infection’ and retaining only objectively confirmed cases. The association between RA and this modified outcome was re-estimated using the same models.

**Table 1.** Baseline patient characteristics before and after propensity score matching (GA+RA vs GA).

	Before matching				After matching			
	No. (%)				No. (%)			
Characteristic	GA+RA(*n* = 5420)	GA(*n* = 3788)	ASD	*p*-Value	GA+RA(*n = 3423)*	GA(*n* = 3423)	ASD	*p*-Value
Age, mean (SD), y	57.62(12.09)	56.85(13.02)	0.064	0.004	56.52(12.37)	56.76(13.07)	0.020	0.430
Female	2815(51.94)	1821(48.07)	0.077	<0.001	1665(48.64)	1694(49.49)	0.017	0.483
Body Mass Index, mean (SD)	23.24(3.17)	23.12(3.35)	0.035	0.104	23.19(3.19)	23.2(3.39)	0.001	0.957
ASA Class 3 or higher	1822(33.62)	1098(28.99)	0.098	<0.001	1006(29.39)	1017(29.71)	0.007	0.771
Preoperative Hemoglobin, mean (SD)	135.86(15.59)	134.14(15.73)	0.110	<0.001	134.74(15.81)	134.71(15.42)	0.002	0.941
Preoperative Albumin, mean (SD)	44.64(3.6)	43.93(4.00)	0.196	<0.001	44.28(3.76)	44.23(3.8)	0.014	0.582
Preoperative steroid use	7(0.13)	5(0.13)	0.008	0.970	4(0.12)	4(0.12)	0.000	1.000
Smoker	1027(18.95)	791(20.88)	0.049	0.022	686(20.04)	690(20.16)	0.003	0.904
Drinker	628(11.59)	451(11.91)	0.010	0.639	390(11.39)	401(11.71)	0.010	0.677
Hypertension	1341(24.74)	857(22.62)	0.049	0.019	776(22.67)	788(23.02)	0.008	0.730
Diabetes	376(6.94)	231(4.26)	0.033	0.110	241(7.04)	219(6.40)	0.025	0.288
COPD	180(3.32)	117(3.09)	0.013	0.535	105(3.07)	107(3.12)	0.003	0.889
Emphysema	307(5.66)	192(5.07)	0.026	0.214	181(5.29)	176(5.14)	0.006	0.786
Preoperative Pulmonary Inflammation	1012(18.67)	728(19.22)	0.014	0.509	647(18.9)	634(18.52)	0.010	0.687
Pulmonary Arterial Hypertension	214(3.95)	145(3.83)	0.006	0.769	133(3.89)	135(3.94)	0.003	0.901
Other Pulmonary Disease	90(1.66)	103(2.72)	0.083	<0.001	74(2.16)	76(2.22)	0.005	0.869
Fluid Balance, mean (SD)	1442.99(417.02)	1489.57(480.36)	0.112	<0.001	1467.72(436.23)	1471.95(462.44)	0.010	0.697
Surgical Duration, mean (SD)	130.18(62.37)	145.4(67.55)	0.244	<0.001	141.43(66.39)	141.82(64.78)	0.006	0.810
Anesthesia Duration, mean (SD)	159.93(62.96)	176.83(69.18)	0.269	<0.001	172.28(67.3)	172.7(66.18)	0.007	0.797
Surgical Type				<0.001				0.957
Wedge Resection	1129(20.83)	725(19.14)	0.042		702(20.51)	692(20.22)	0.007	
Segmentectomy	527(9.72)	277(7.31)	0.081		276(8.06)	269(7.86)	0.007	
Lobectomy	3725(68.73)	272071.81)	0.066		2411(70.44)	2423(70.79)	0.008	
Sleeve resection	17(0.31)	19(0.5)	0.034		14(0.41)	15(0.44)	0.005	
Bilobectomy	3(0.06)	6(0.16)	0.044		3(0.09)	2(0.06)	0.012	
Pneumonectomy	19(0.35)	41(1.08)	0.124		17(0.50)	22(0.64)	0.025	
Surgical Approach			0.480	<0.001			0.039	0.200
Thoracotomy	195(3.6)	475(12.54)			190(5.55)	215(6.28)		
Video-assisted Thoracoscopic Surgery	5225(96.4)	3313(87.46)			3233(94.45)	3208(93.72)		
Use of Blood Product	13(0.24)	7(0.18)	0.011	0.577	8(0.23)	7(0.20)	0.006	0.796
Use of Muscle Relaxants	733(13.52)	591(15.60)	0.061	0.005	526(15.37)	515(15.05)	0.009	0.711

Abbreviations: GA, general Anesthesia; GA+RA, general Anesthesia combined with regional Anesthesia; ASD, absolute standardized difference; BMI, body mass index (calculated as weight in kilograms divided by height in meters squared); ASA, American Society of Anesthesiologists Physical Status classification; COPD, chronic obstructive pulmonary disease; SD, Standard deviation.

**Table 2.** Baseline patient characteristics before and after propensity score matching (GA+PRA vs GA).

	Before matching				After matching			
	No. (%)				No. (%)			
Characteristic	GA+PRA(*n* = 5246)	GA(*n* = 3788)	ASD	*p*-value	GA+PRA(*n* = 3369)	GA(*n* = 3369)	ASD	*p*-value
Age, mean (SD), y	57.6(12.14)	56.85(13.02)	0.0622	0.005	56.8(12.26)	56.78(13.15)	0.0021	0.935
Female	2730(50.04)	1821(48.07)	0.0794	<0.001	1658(49.21)	1665(49.42)	0.0042	0.865
Body Mass Index, mean (SD)	23.24(3.18)	23.12(3.35)	0.0366	0.093	23.24(3.18)	23.18(3.38)	0.0198	0.432
ASA Class 3 or higher	1765(33.64)	1098(28.99)	0.0986	<0.001	1027(30.48)	995(29.53)	0.0201	0.395
Preoperative Hemoglobin, mean (SD)	135.81(15.60)	134.14(15.73)	0.1071	<0.001	134.79(15.74)	134.72(15.39)	0.0048	0.845
Preoperative Albumin, mean (SD)	44.63(3.58)	43.93(4.00)	0.1968	<0.001	44.27(3.67)	44.23(3.81)	0.0129	0.612
Preoperative steroid use	7(13.34)	5(0.13)	0.0004	0.985	3(0.8)	4(0.12)	0.0081	0.705
Smoker	998(19.02)	791(20.88)	0.0473	0.029	670(19.89)	685(20.33)	0.0113	0.648
Drinker	608(11.59)	451(11.91)	0.0099	0.645	390(11.58)	400(11.87)	0.0093	0.705
Hypertension	1299(24.76)	857(22.62)	0.0495	0.019	785(23.30)	789(23.42)	0.0028	0.908
Diabetes	367(7.00)	231(6.10)	0.0352	0.090	225(6.68)	211(6.26)	0.0166	0.488
COPD	176(3.35)	117(3.09)	0.0148	0.481	99(2.9)	112(3.32)	0.0214	0.363
Emphysema	300(5.72)	192(5.07)	0.0280	0.179	172(5.1)	179(5.31)	0.0089	0.701
Preoperative Pulmonary Inflammation	982(18.72)	728(19.22)	0.0128	0.550	622(18.46)	632(18.76)	0.0076	0.754
Pulmonary Arterial Hypertension	207(3.95)	145(3.83)	0.0061	0.775	139(4.1)	137(4.07)	0.0030	0.902
Other Pulmonary Disease	84(1.60)	103(2.72)	0.0891	<0.001	74(2.2)	75(2.22)	0.0024	0.934
Fluid Balance	1440.91(416.34)	1489.57(480.36)	0.1169	<0.001	1464.08(429.45)	1470.61(460.30)	0.0157	0.547
Surgical Duration	130.02(62.60)	145.4(67.55)	0.2456	<0.001	141.17(66.10)	140.77(64.31)	0.0065	0.798
Anesthesia Duration	159.56(63.01)	176.83(69.18)	0.2741	<0.001	172.06(66.67)	171.51(65.75)	0.0088	0.730
Surgical Type				<0.001				0.999
Wedge Resection	1113(21.22)	725(19.14)	0.0514		680(20.18)	688(20.42)	0.0058	
Segmentectomy	505(9.63)	277(7.31)	0.0784		262(7.78)	265(7.87)	0.0030	
Lobectomy	3592(68.47)	2720(71.81)	0.0718		2396(71.12)	2387(70.85)	0.0057	
Sleeve resection	16(0.30)	19(0.5)	0.0357		12(0.36)	12(0.36)	0.0000	
Bilobectomy	2(0.04)	6(0.16)	0.0616		2(0.05)	2(0.06)	0.0000	
Pneumonectomy	18(0.34)	41(1.08)	0.1264		17(0.50)	15(0.45)	0.0102	
Surgical Approach			0.5028	<0.001			0.0409	0.184
Thoracotomy	179(3.41)	475(12.54)			175(5.19)	200(5.94)		
Video-assisted Thoracoscopic Surgery	5067(96.59)	3313(87.46)			3194(94.81)	3169(94.06)		
Use of Blood Product	13(0.25)	7(0.18)	0.0127	0.529	4(0.12)	5(0.15)	0.0060	0.739
Use of Muscle Relaxants	700(13.34)	591(15.60)	0.0664	0.002	509(15.11)	505(14.99)	0.0035	0.892

Abbreviations: GA, general anesthesia; GA+PRA, general anesthesia combined with peripheral regional anesthesia; ASD, absolute standardized difference; BMI, body mass index (calculated as weight in kilograms divided by height in meters squared); ASA, American Society of Anesthesiologists Physical Status classification; COPD, chronic obstructive pulmonary disease; SD, Standard deviation.

The associations between the anesthesia type and binary outcomes were determined by calculating HRs and 95% confidence intervals (CIs) with a Cox proportional hazards regression model, whereas the associations with postoperative hospital stay and hospitalization cost were evaluated using generalized linear models with an inverse Gaussian distribution. Given the numerous secondary outcomes, Bonferroni correction [24] was applied to control the type I error rate when multiple testing was performed, and the reported *p* values for secondary outcome analyses were all Bonferroni-corrected.

All statistical analyses were conducted using SAS statistical software, version 9.4 (SAS Institute, Inc.) and R software, version 4.4.1 (R Foundation for Statistical Computing). A two-sided *p* value of <0.05 was considered to indicate statistical significance, if not otherwise specified.

## Results

**Figure 1.**
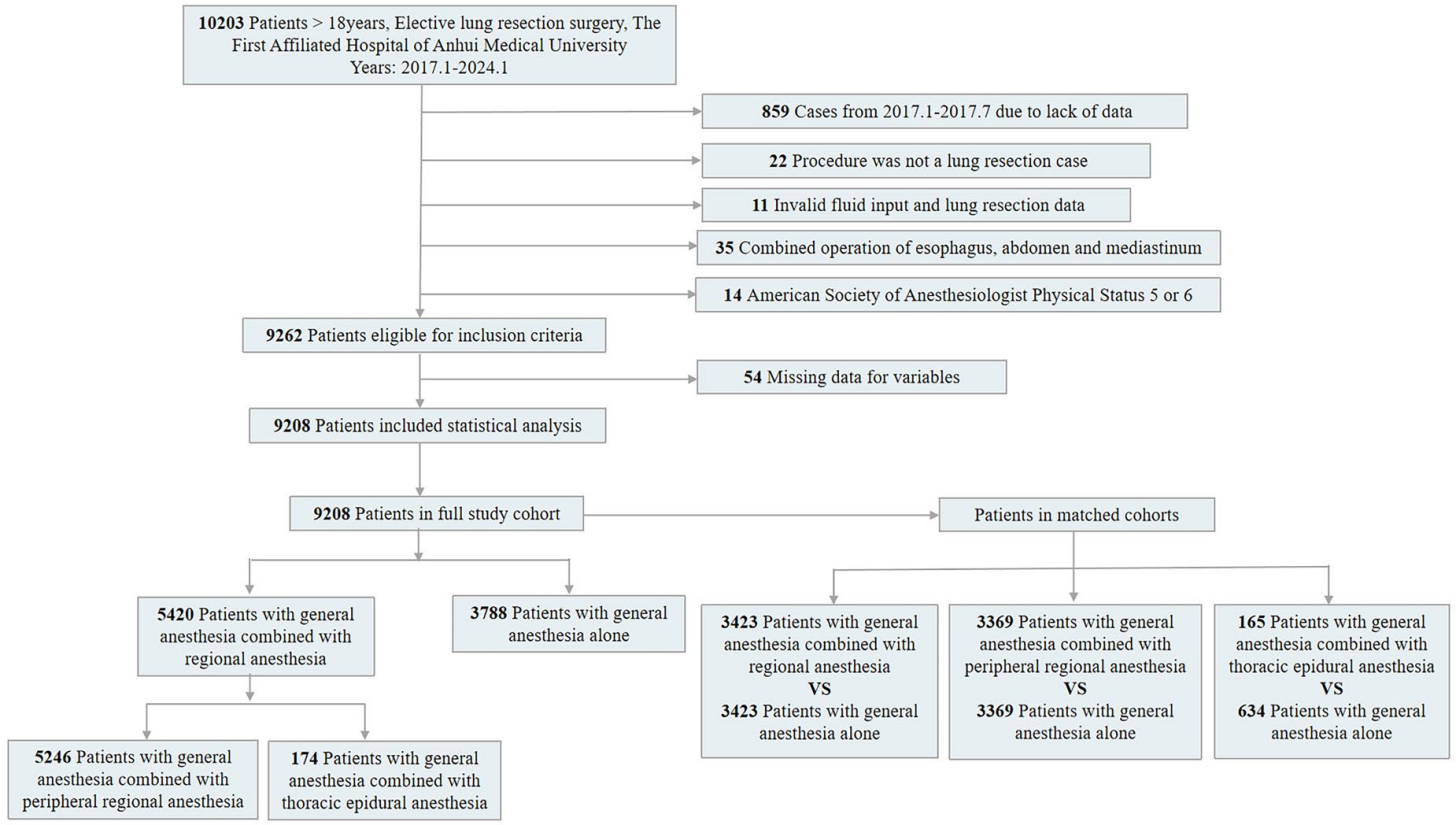
Selection process of participants for the study.

A total of 10,203 patient records were collected, and 941 cases were excluded. The remaining 9208 patients who underwent lung resection surgery were included in this study. Among the 9208 patients, 5420 received a combination of general and regional anesthesia (5246 received general anesthesia combined with peripheral regional anesthesia, and 174 received general anesthesia combined with thoracic epidural anesthesia), and 3788 received GA alone (Figure 1).

Table S1 provides a detailed description of respiratory symptoms and the diagnosis of PPCs. Among the 9208 patients, 34.9% had PPCs. Among PPCs, the incidence of suspected pulmonary infection was the highest (28.4%), followed by pneumothorax (9.5%), atelectasis (5.6%), pleural effusion (4.4%), respiratory failure (1.4%), bronchospasm (0.6%), and aspiration pneumonitis (0.3%).

We conducted pairwise comparisons between the GA group and each of the intervention groups (GA + RA, GA + PRA, and GA + EA), followed by propensity score matching (Figure 1). After propensity score matching, among the three groups, 3423 patients in the GA group were successfully matched with 3423 patients in the GA + RA group; 3369 patients in the GA group were matched with 3369 patients in the GA + PRA group; and 634 patients in the GA group were successfully matched with 165 patients in the GA + EA group. No significant differences were noted in patient characteristics between the total population and the PS-matched population (Tables 1–2, S3). Tables 1, 2, and S3 show the baseline characteristics of the six cohorts (GA group vs. GA + RA group, GA group vs. GA + PRA, and GA group vs. GA+ EA group, respectively) before and after propensity score matching. For the other subgroup comparisons (GA + PVB vs. GA, GA + SAPB vs. GA, GA + ICNB vs. GA, and GA + Others vs. GA), the balance between the characteristics was similarly satisfactory after matching (see Supplementary Table S7). All the cohorts were well-balanced across all the observed features, with no ASMD exceeding 0.1 and the majority of the ASMD values not exceeding 0.01. Before matching for the overall cohort, a total of 5420 (58.86%) patients received GA combined with RA, and 3788 (41.14%) received GA alone. The mean age of patients in the GA+ RA cohort was 57.62 years (SD, 12.09), with 51.94% being female, and it was 56.85 years (SD, 13.02) in the GA cohort, with 48.07% being female. The mean age of patients in the GA + RA cohort was 56.52 years (SD, 12.37), and it was 56.76 years (SD, 13.07) in the GA cohort. The GA + RA cohort included 1665 (48.64%) female patients, and the GA cohort included 1694 (49.49%) female patients (Table 1).

**Table 3.** Secondary outcomes in patients who received GA vs GA+PRA in the propensity score matched cohort.

	GA+PRA (*n* = 3369)	GA (*n* = 3369)			
Postoperative Outcomes	No. (%)	No. (%)	HR (95% CI)^†^	*p*-value	Adjusted *p*-value^‡^
**MACE**	178(5.27)	193(5.71)	0.916(0.747–1.123)	0.399	0.501
New Cardiac Arrhythmia	146(4.32)	134(3.97)	1.081(0.855–1.367)	0.516	0.550
Acute Myocardial Infarction	26(0.76)	46(1.36)	0.567(0.350–0.918)	0.021	0.080
Congestive Heart Failure.	14(0.41)	34(1.01)	0.415(0.223–0.774)	0.006	0.032
Angina	5(0.15)	16(0.47)	0.317(0.116–0.868)	0.025	0.080
Postoperative Myocardial Injury	11(0.33)	15(0.44)	0.728(0.334–1.588)	0.425	0.500
Non-fatal cardiac arrest	1(0.03)	5(0.15)	0.220(0.025–1.903)	0.169	0.346
Hypotension	52(1.54)	69(2.04)	0.749(0.523–1.075)	0.117	0.312
Slow Heart Rate	51(1.51)	40(1.18)	1.271(0.840–1.924)	0.256	0.455
Pain	1883(55.73)	2096(62.03)	0.897(0.842–0.954)	<0.001	<0.001
Opioid consumption					
24MME	43.8(38.1–50.9)	60.9(55.3–69.1)		<0.001^§^	<0.001^§^
48MME	88.2(76.3–101.6)	120.4(108.1–135.6)			
Total MME	131.3(112.5–151.9)	176.9(160–199)			
ARI	36(1.06)	43(1.27)	0.839(0.538–1.308)	0.438	0.501
Unplanned Tracheal Ontubation	11(0.33)	18(0.53)	0.680(0.316–1.462)	0.323	0.501
Unexpected ICU Admission	14(0.41)	21(0.62)	0.721(0.361–1.438)	0.353	0.501
Automatic Discharge	4(0.12)	7(0.21)	5.282(0.482–57.892)	0.173	0.346
Hospitalization Costs, median (IQR)	40918.8(34339.4–47578.0)	40523.5(33339.8–46948.0)		0.947^§^	0.947^§^
Postoperative Hospital Stay, median (IQR)	4.67(3.7–5.97)	4.8(3.7–6.6)		<0.001^§^	<0.001^§^

Abbreviations: GA, general anesthesia; RA, regional anesthesia; MACE, major adverse cardiac events; ARI, acute renal injury; ICU, intensive care unit; IQR, interquartile range.

^†^ *The GA group is the reference group (HR = 1.00). HR < 1 indicates a lower risk in the GA+RA group; HR > 1 indicates a higher risk in the GA+RA group.*.

^‡^ *p-Values were adjusted for multiple testing using the Bonferroni method.

^§^ *p*-Value for continuous outcomes (costs, length of stay) derived from Wilcoxon rank-sum test.

### Primary outcomes and subgroup analysis

The incidence rates of PPCs were 35.5% (1216/3423) in the GA + RA group versus 37.9% (1296/3423) in the GA group. Compared with the GA group, the GA + RA group showed a nonsignificant trend toward a lower incidence of PPCs (HR, 0.93; 95% CI, 0.86–1.01; *p* = 0.078) (Figure S1, Table S5). Similarly, relative to the GA group, the incidence of postoperative pulmonary complications (PPCs) was not significantly lower in the GA + PRA group (HR, 0.95; 95% CI, 0.879–1.03; *p* = 0.221) (Figure S2; Table S5) or the GA + EA group (HR, 0.85; 95% CI, 0.62–1.15; *p* = 0.25) (Figure S3; Table S5). To explore the potential differential effects of specific PRA techniques, we performed stratified analyses in which each PRA subtype was compared with GA alone. The cohort included patients who underwent PVB (*n* = 281, 5.36%), SABP (*n* = 1982, 37.78%), ICNB (*n* = 2118, 40.37%), and other or combined techniques (*n* = 865, 16.49%). As shown in Table S5, PVB and SAPB were associated with significant reductions in the incidence rates of PPCs (HR, 0.77; 95% CI, 0.61–0.98; *p* = 0.03 and HR, 0.81; 95% CI, 0.73–0.91; *p* ≤ 0.001), whereas the estimates for ICNB and others did not reach statistical significance (*p* = 0.086 and *p* = 0.362, respectively). However, the sample sizes of both the PVB group and the other groups were limited. The findings warrant validation in larger-scale, adequately powered clinical trials. These exploratory findings require confirmation in future studies.

**Figure 2.**
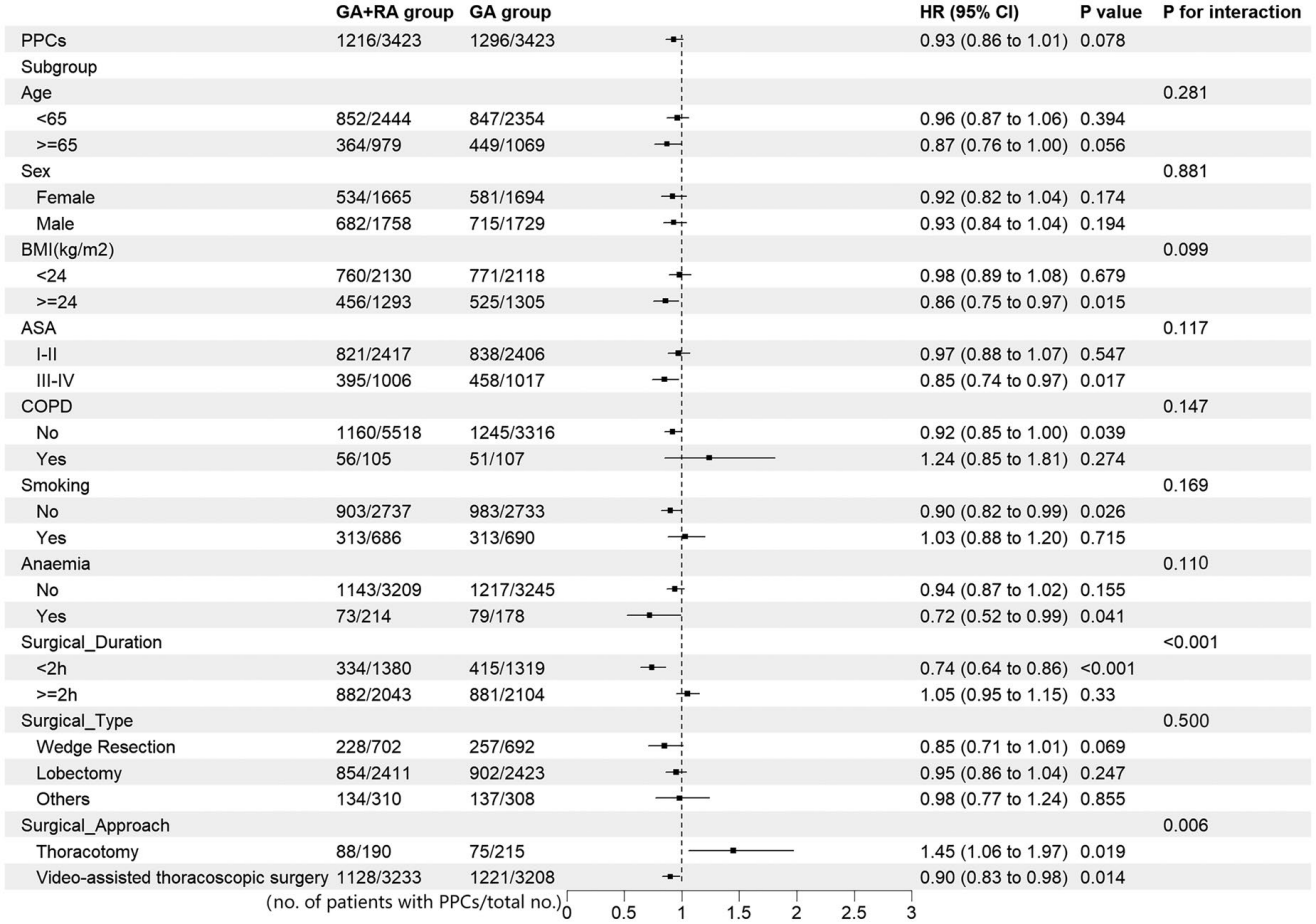
Primary outcome in the propensity score-matched cohort and subgroup-specific associations of GA vs GA+RA. Abbreviations: GA, general anesthesia; GA+RA, general anesthesia in conjunction with regional anesthesia; PPCs, postoperative pulmonary complications; HR, hazard ratio; BMI, body mass index; ASA, American Society of Anesthesiologists Physical Status classification; COPD, Chronic obstructive pulmonary disease.

Figure 2 presents the HRs for subgroups stratified by age, sex, BMI, ASA status, smoking status, anemia presence, surgical duration, surgical type, and surgical approach. Significant interactions were observed for surgical duration and surgical approach on PPCs (P for interaction < 0.001 and *p* for interaction = 0.006, respectively). Specifically, among patients with a surgical duration of <2 h (HR, 0.74; 95% CI, 0.64–0.86) and those who underwent video-assisted thoracoscopic surgery (HR, 0.90; 95% CI, 0.83–0.98), a lower incidence of PPCs was observed in the GA + RA group than in the GA group (*p* < 0.001 and *p* = 0.014, respectively). All subgroup analyses were exploratory (not prespecified in the study protocol). The workflow of this study is summarized in the graphical abstract.

### Secondary outcomes

#### GA + PRA vs. GA

After propensity score matching, 3369 patients in the GA + PRA group and 3369 patients in the GA group were successfully matched. Compared with the GA group, the GA + PRA group exhibited lower incidence rates of postoperative heart failure (adjusted *p* = 0.032), pain (adjusted *p* < 0.001), and opioid consumption (adjusted *p* < 0.001) and a shorter postoperative hospital stay (Table 3).

#### GA + EA vs. GA

Among the 5420 patients in the GA + RA group, 174 received GA combined with EA. After 1:4 propensity score matching, 165 patients in the GA + EA group and 634 patients in the GA group were successfully matched (Table S4). Regarding secondary outcomes, the GA + EA group exhibited a higher incidence rate of hypotension (adjusted *p* = 0.035) and a lower incidence rate of opioid consumption (adjusted *p* < 0.001) and pain (adjusted *p* < 0.001). However, no significant differences were observed in the other outcomes between the two groups (Table S4).

### Sensitivity analysis

In the sensitivity analysis excluding suspected pulmonary infection cases and retaining only objectively confirmed cases based on the PPC definition, the association between RA and the incidence of PPCs remained consistent with the primary findings. Compared with GA alone, the GA + RA combination was associated with the modified PPC outcome (HR, 0.97; 95% CI, 0.89–1.05; *p* = 0.48) (Table S6). These results were similar in direction and magnitude to those of the primary analysis (HR = 0.93).

## Discussion

To the best of our knowledge, this is the largest study to investigate the associations between the incidence of PPCs and RA in patients who underwent lung resection surgery. In this cohort study, no significant difference was observed in the primary endpoint of PPCs between patients who received GA combined with RA and those who received GA alone. However, in the subgroups with a surgery duration of ≤2 h and the use of a thoracoscopic surgical approach, patients who received GA alone exhibited a higher risk of PPCs than those who received GA combined with RA did. Combined RA (PRA or EA both) with GA was associated with a lower incidence of pain (*p* < 0.001), but combined EA with GA was associated with a higher incidence of hypotension (*p* = 0.005). Combined PRA with GA was associated with a significant reduction of 58% in the heart failure rate (HR = 0.42, *p* = 0.006).

PPCs are frequent and potentially severe adverse events following thoracic surgery [25]. The etiology of PPCs is multifactorial, primarily encompassing postoperative respiratory muscle dysfunction, ventilation–perfusion mismatch, diminished cough efficacy, accumulation of respiratory secretions, reduced tidal volume, decreased functional residual capacity and vital capacity, and postoperative pain [26]. Surgical pain and GA induction can impair lung function, and to a lesser extent, these associations may persist into the postoperative period [26]. Some studies have suggested that combined RA, particularly thoracic epidural anesthesia (TEA), with GA predominantly exerts lung-protective effects. The underlying mechanism may, at least in part, involve the superior analgesic properties of RA, which facilitate early patient mobilization and optimize the postoperative lung volume; these favorable outcomes have been shown to reduce PPCs and enhance patient recovery after surgery [10,27–29]. However, some studies have yielded contrasting results. Studies have indicated that despite being associated with lower pain scores and reduced opioid consumption, RA, regardless of the use of TEA or peripheral regional anesthesia, does not reduce the incidence of PPCs [30–32]. Currently, there is a growing consensus that ERAS programs, which incorporate advancements in surgical techniques, respiratory physiotherapy, early mobilization, and prophylactic antibiotics, have diminished the relative advantage of RA in improving postoperative pulmonary outcomes over the past few decades [33]. In this study, no statistically significant difference was found in the incidence of PPCs between patients receiving general anesthesia combined with RA (PRA or EA) and patients receiving only general anesthesia.

Importantly, in our subgroup analysis, the influence of the surgical duration (P for interaction < 0.001) on the outcomes was significantly different. Additionally, the point estimate (HR = 0.74) in the <2-hour subgroup may overstate the true effect because of its exploratory nature and winner’s curse bias; a more conservative estimate might be closer to HR = 0.83. Although these results suggest the role of RA in PPC development in select subgroups, definitive recommendations can be made only with confirmation in adequately powered, prospective trials. In another subgroup analysis stratified by surgical approach, the effect of regional anesthesia on PPC onset differed between the thoracoscopic and open surgery subgroups. However, following statistical correction for multiple comparisons, the observed differences did not remain statistically significant (*p* > 0.05). Numerous studies have confirmed that various regional block techniques are advantageous for postoperative analgesia and patient outcomes following thoracoscopic surgery [34,35]. However, owing to the extensive trauma and severe postoperative pain associated with thoracotomy, only TEA and paravertebral block can effectively alleviate pain, whereas other blocking techniques have limited efficacy [34,36]. The adverse effects of TEA on motor function and sympathetic innervation, along with its detrimental impact on diaphragm function, collectively contribute to postoperative pulmonary dysfunction [37]. Furthermore, given the small sample size of the open surgery subgroup (*n* = 405) and the inherent complexity of open surgery with numerous influencing factors, the relationship between regional analgesia and PPCs requires further investigation. However, all subgroup analyses were exploratory (not prespecified in the study protocol) and should be interpreted as hypothesis-generating.

Furthermore, regarding secondary outcomes, our study revealed significant and previously underappreciated differences in the therapeutic effects of the various types of RA techniques (PRA or EA) when used in combination with GA. A stratified analysis revealed divergent safety and efficacy profiles that fundamentally altered the interpretation of our results and their clinical implications. The most plausible explanation for this discrepancy is the distinct physiological effects of neuraxial versus peripheral nerve blockade. EA induces profound and extensive sympathetic blockade, leading to significant vasodilation and reduced systemic vascular resistance. This mechanism directly accounts for the significantly higher incidence of hypotension in our GA + EA cohort [10,38]. Furthermore, this sympathetic blockade can extend to cardiac accelerator fibers (T1–T4), potentially impairing the ability of the heart to compensate through tachycardia [38]. Although EA provides superior analgesic effects, which theoretically could benefit pulmonary function by enabling deeper breathing and coughing, these potential advantages appear to be counterbalanced, or even outweighed, by the resulting hemodynamic instability. The resulting hypotension and the potential for fluid overload during its management may promote pulmonary edema and ventilation–perfusion mismatching, nullifying any net benefit in preventing PPCs [39,40]. However, the small sample size of the GA + EA subgroup may have limited the statistical power to detect clinically meaningful differences in the outcomes. Thus, the nonsignificant results—such as the absence of a statistically significant reduction in PPCs—should be interpreted cautiously given the potential for type II error. Larger, adequately powered studies including patients receiving epidural anesthesia should be conducted to clarify its impact on clinical outcomes.

In contrast, PRA techniques provide highly targeted analgesia with minimal systemic hemodynamic consequences. Their sympathetic blockade is confined to the innervated limb, resulting in no significant increase in hypotension, as confirmed by our analysis. The benefits of PRA likely result from a multifaceted mechanism: excellent site-specific pain control leading to a reduction in systemic opioid consumption, attenuation of the surgical stress response, and facilitation of early patient mobilization [41]. The significant reduction in the heart failure rate in the GA + PRA group is notable, and the reduction may be attributed to the reduced catecholamine surge and myocardial oxygen demand caused by improved analgesic effects, in addition to the avoidance of the fluid shift often associated with managing EA-induced hypotension.

These findings have immediate clinical relevance. For patients at high risk of PPCs or hemodynamic instability, PRA may be the preferred adjunct to GA because of its favorable safety profile and significant benefits. Although EA is a powerful technique, meticulous hemodynamic management is required. Thus, this technique is best applied in specific scenarios where its unique benefits are paramount. Future studies must stratify outcomes by RA type. To guide more precise perioperative care, randomized trials should directly compare modern EA management strategies with advanced PRA techniques in specific surgical and patient populations.

Additionally, cardiac complications following thoracic surgery warrant significant attention [42]. Currently, the findings regarding the effects of RA on postoperative cardiovascular complications remain inconsistent. Some meta-analyses, primarily comprising studies on vascular surgery, have demonstrated significant reductions in cardiac morbidity and myocardial infarction (MI) rates with epidural techniques [43]. However, other studies have not revealed a significant association between epidural analgesia and the incidence of cardiovascular complications [44,45]. In this study, RA reduced the incidence of postoperative adverse cardiac events such as heart failure. The primary underlying mechanism may involve improved postoperative pain management and reduced stress responses caused by pain stimulation. Further research is needed to elucidate the specific reasons. Additionally, RA has been shown to reduce the length of postoperative hospital stay and improve overall prognosis in patients who underwent thoracic surgery.

### Limitations

This study has several limitations. First, the small sample sizes of the thoracotomy surgery subgroup and the GA + EA subgroup limit the generalizability of the results, especially for the GA + EA subgroup. This limited sample size resulted in reduced statistical power to detect potentially meaningful differences in the outcomes between the GA + EA and GA alone groups. Future studies with larger sample sizes of patients receiving epidural anesthesia are needed to more definitively evaluate its effects on the incidence of postoperative pulmonary complications. Second, detailed information on intraoperative pain scores and related ERAS protocol elements was lacking from the data sources. While all blocks were single-injection-administered according to the departmental protocol (aimed at providing thoracic analgesia), we cannot determine whether certain techniques were more effective than others were. However, the robust reduction in opioid consumption is a unified, objective mechanism that is likely common to all effective regional techniques. Notably, a formal ERAS protocol was not uniformly implemented at our institution during the study period. Although some ERAS elements may have been applied at clinicians’ discretion, their adherence could not be systematically captured. Routine postoperative physiotherapy is standard practice at our institution, but detailed data on the timing, intensity, and adherence to physiotherapy protocols were not available in our database. These represent potential sources of unmeasured confounding. Thus, future prospective studies should aim to systematically capture these variables. Third, as this was a single-center retrospective study conducted at a large teaching hospital, risks of misclassification bias and selection bias are prevalent. Despite robust statistical techniques, residual confounding and indication bias remain inherent challenges in cohort studies. Nonetheless, to the best of our knowledge, this is the largest study to date examining the associations between the incidence of PPCs and regional anesthesia in patients who underwent lung resection surgery. Importantly, our subgroup analysis provide clinically significant insights. The results suggest that the use of RA in thoracoscopic surgery with short operation times may be more advantageous for reducing postoperative pulmonary complications.

## Conclusions

This study revealed no statistically significant difference in the incidence of PPCs between patients who received the general anesthesia combined with regional anesthesia (GA + RA) and those who received general anesthesia alone (GA). However, a clinically meaningful reduction in the incidence of PPCs was observed in prespecified subgroups—specifically, patients who underwent thoracoscopic surgery with an operative duration of ≤2 h. In this subgroup, PRA may be preferred over EA to ensure analgesic efficacy while avoiding hemodynamic fluctuations. These findings suggest the selective benefit of regional anesthesia (RA) in well-defined patient subgroups. However, these findings remain as hypothesis-generating and warrant validation in adequately powered studies in future.

## Supplementary Material

Supplemental Material

Supplemental Material

Supplemental Material

Supplemental Material

Supplemental Material

revised_supplemental_tables clean file.docx

## Data Availability

The datasets generated and analysed during the current study are available from the corresponding author on reasonable request.
