# Supplementary material for: The association between regional anesthesia and postoperative pulmonary complications following lung resection surgery: a hospital-based, retrospective cohort study
Source: Ann Med. 2026 May 26;58(1):2677995. doi: 10.1080/07853890.2026.2677995 (PMC13215415; doi:10.1080/07853890.2026.2677995)
Supplement: Supplemental Material [file IANN_A_2677995_SM3054.docx]

**Legends for supplementary figures and tables**

Figure S1. Cumulative Hazards of Postoperative Pulmonary Complications Among Patients Who Received GA+RA or GA.

Abbreviations: GA, general anesthesia; GA+RA, general anesthesia combined with regional anesthesia; PPCs, postoperative pulmonary complications.

Figure S2. Cumulative Hazards of Postoperative Pulmonary Complications Among Patients Who Received GA+PRA or GA.

Abbreviations: GA, general anesthesia; GA+PRA, general anesthesia combined with peripheral regional anesthesia; PPCs, postoperative pulmonary complications; HR, hazard ratio.

Figure S3. Cumulative Hazards of Postoperative Pulmonary Complications Among Patients Who Received GA+EA or GA

Abbreviations: GA, general anesthesia; GA+EA, general anesthesia combined with epidural anesthesia; PPCs, postoperative pulmonary complications; HR, hazard ratio.

Figure S4. A love plot.

Abbreviations: BMI, body mass index; COPD, chronic obstructive pulmonary disease.
