# Supplementary figures and images for: The association between regional anesthesia and postoperative pulmonary complications following lung resection surgery: a hospital-based, retrospective cohort study
Source: Ann Med. 2026 May 26;58(1):2677995. doi: 10.1080/07853890.2026.2677995 (PMC13215415; doi:10.1080/07853890.2026.2677995)

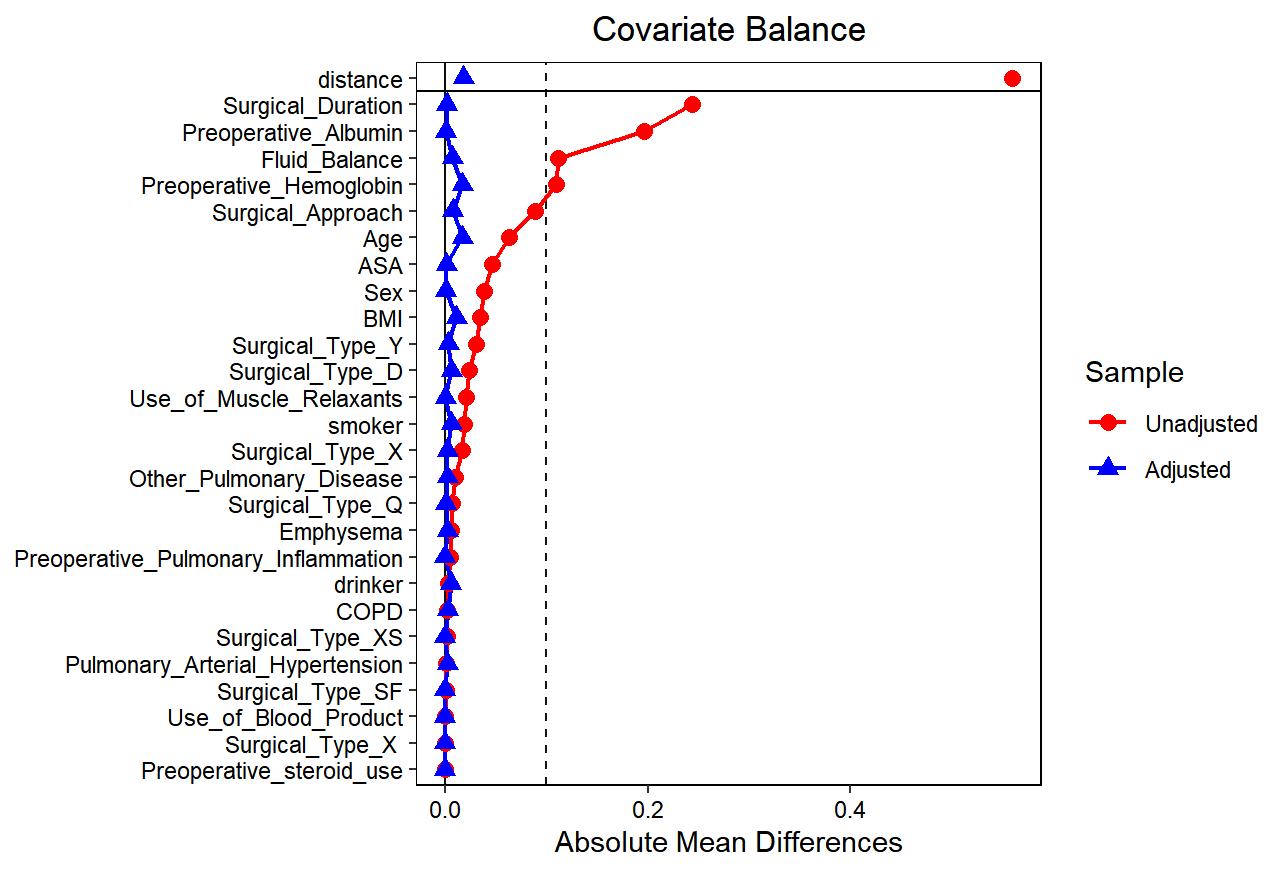

Supplement: Supplemental Material [file IANN_A_2677995_SM3052.tif]

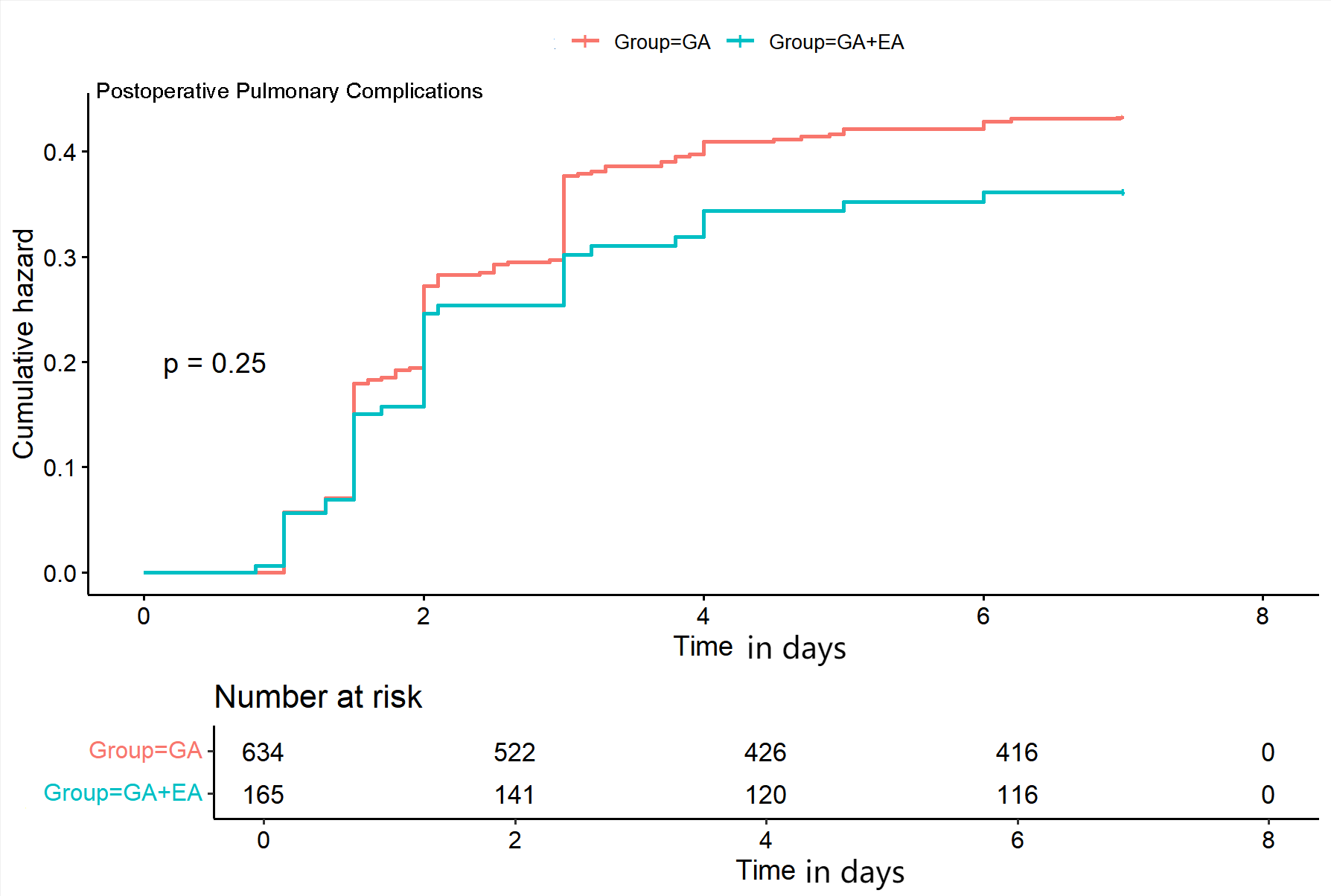

Supplement: Supplemental Material [file IANN_A_2677995_SM3051.tif]

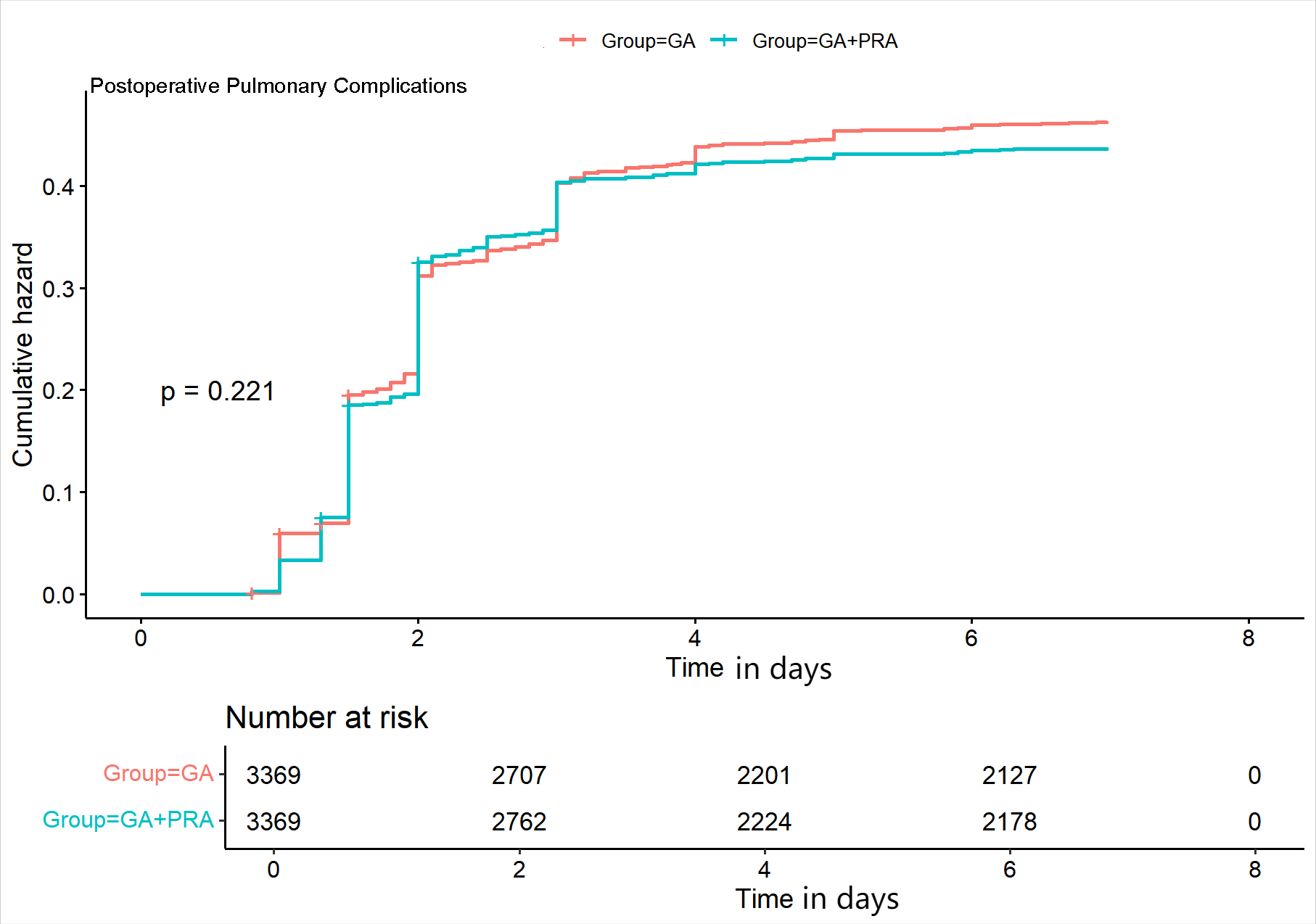

Supplement: Supplemental Material [file IANN_A_2677995_SM3050.tif]

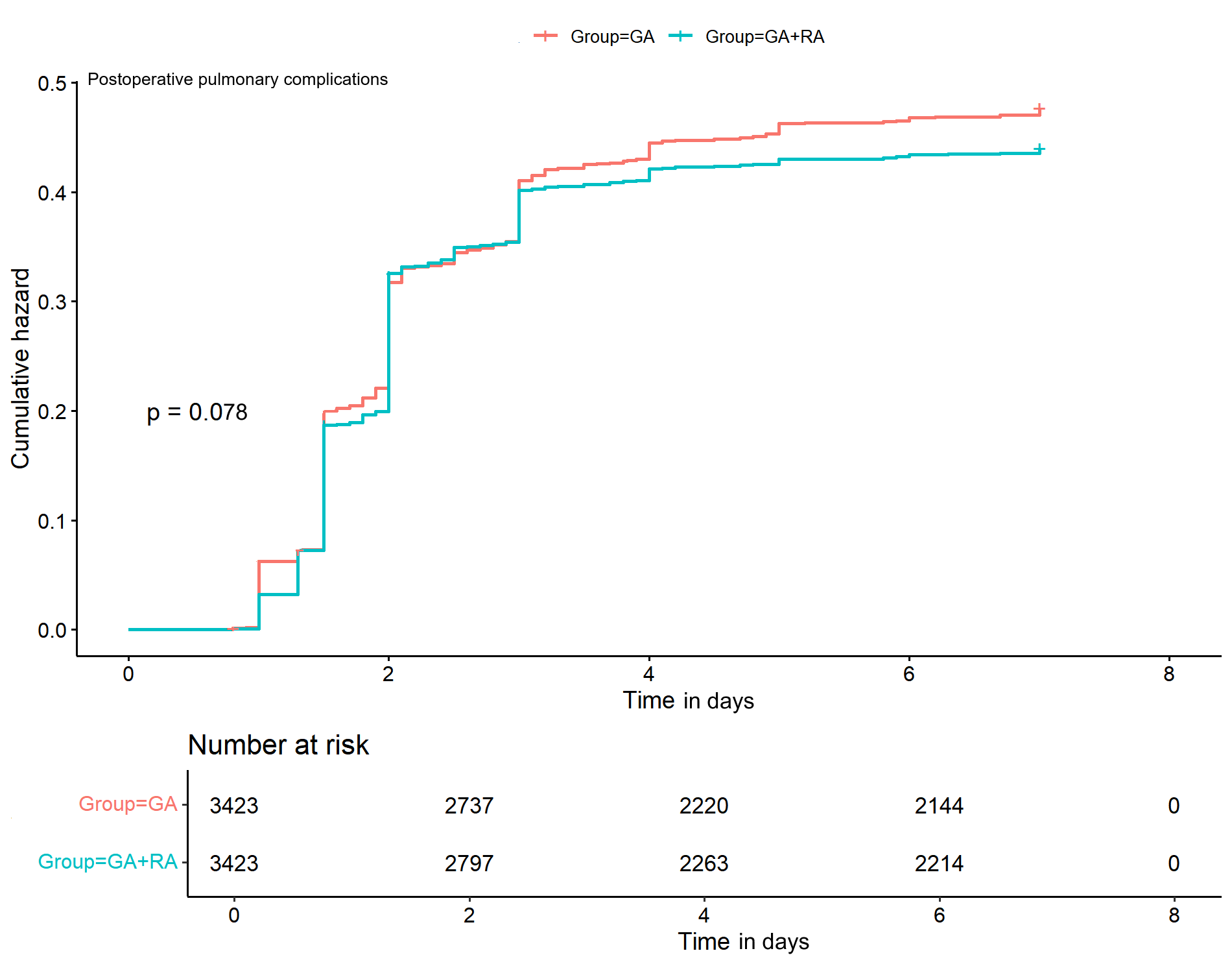

Supplement: Supplemental Material [file IANN_A_2677995_SM3049.tif]
