# Supplementary material for: The association between regional anesthesia and postoperative pulmonary complications following lung resection surgery: a hospital-based, retrospective cohort study
Source: Ann Med. 2026 May 26;58(1):2677995. doi: 10.1080/07853890.2026.2677995 (PMC13215415; doi:10.1080/07853890.2026.2677995)
Supplement: revised_supplemental_tables clean file.docx [file IANN_A_2677995_SM2062.docx]

**Supplemental Online Content**

**Table S1. Definitions of postoperative pulmonary complications and their incidences observed in 9208 patients.**

|  | Clinical Syndrome | Incidence |
| --- | --- | --- |
| Respiratory failure | Postoperative PaO2 < 8 kPa (60 mmHg) on room air, a PaO2:FI02 ratio <40 kPa (300 mmHg) or arterial oxyhaemoglobin saturation measured with pulse oximetry < 90% and requiring oxygen therapy | 1.40% |
| Suspected pulmonary infection | General signs of infection, at least one of the following criteria: patient receives antibiotics, core body temperature >38°C, leucocytosis >12000 cells per μL; and signs of an infection of pulmonary origin, at least one of the following criteria: new or changed sputum, or new or changed lung opacity on chest x-ray when clinically indicated | 28.40% |
| Pleural effusion | Chest radiograph demonstrating blunting of the costophrenic angle, loss of sharp silhouette of the ipsilateral hemidiaphragm in upright position, evidence of displacement of adjacent anatomical structures or (in supine position) a hazy opacity in one hemithorax with preserved vascular shadows | 4.40% |
| Atelectasis | Lung opacification with a shift of the mediastinum, hilum or hemidiaphragm toward the affected area, and compensatory over-inflation in the adjacent non-atelectatic lung | 5.60% |
| Pneumothorax | Air in the pleural space with no vascular bed surrounding the visceral pleura | 9.50% |
| Bronchospasm | Newly detected expiratory wheezing treated with bronchodilators | 0.60% |
| Aspiration pneumonitis | Acute lung injury after the inhalation of regurgitated gastric contents | 0.30% |
| Primary outcomes | Postoperative pulmonary complications (at least one character) | 34.90% |

Relevant symptoms align with those specified in the European Perioperative Clinical Outcome (EPCO) definitions.^17^ Abbreviations: PaO2, partial pressure of oxygen in arterial blood; FiO2, fractional concentration of oxygen in inspired air.

**Table S2. Major Adverse Cardiac Events (MACE) definition.**

| New Cardiac Arrhythmia | ECG evidence of atrial flutter, atrial fibrillation, or second- or third-degree atrioventricular conduction block. |
| --- | --- |
| Acute Myocardial Infarction | Increase and gradual decrease in troponin level or a faster increase and decrease of creatine kinase isoenzyme as markers of myocardial necrosis in the company of at least one of the following: ischaemic symptoms, abnormal Q waves on the ECG, ST segment elevation or depression; coronary artery intervention (e.g. coronary angioplasty) or a typical decrease in an elevated troponin level detected at its peak after surgery in a patient without a documented alternative explanation for the troponin elevation. |
| Congestive Heart Failure. | New in-hospital signs or symptoms of dyspnoea or fatigue, orthopnoea, paroxysmal nocturnal dyspnoea, increased jugular venous pressure, pulmonary raˆles on physical examination, cardiomegaly or pulmonary vascular engorgement. |
| Angina | Dull diffuse substernal chest discomfort precipitated by exertion or emotion and relieved by rest or glyceryl trinitrate. |
| Postoperative Myocardial Injury | an elevation in cTn-levels exceeding the 99th percentile， without clinical signs or symptoms |
| Non-fatal cardiac arrest | An absence of cardiac rhythm or presence of chaotic rhythm requiring any component of basic or advanced cardiac life support. |

MACEs are defined as one or more of the Following aspects, in accordance with the European Perioperative Clinical Outcome (EPCO) definitions.^17^ Abbreviations: MACE, major adverse cardiac events; ECG, Electrocardiogram.

**Table S3. Baseline Patient Characteristics Before and After Propensity Score Matching (GA+EA vs GA).**

|  | Before matching |  |  |  |  | After matching |  |  |  |
| --- | --- | --- | --- | --- | --- | --- | --- | --- | --- |
|  | No. (%) |  |  |  |  | No. (%) |  |  |  |
| Characteristic | GA+EA(*n=174*) | GA(*n=3788*) | ASD^†^ | *P-Value* |  | GA+EA(*n=165*) | GA(*n=634*) | ASD^†^ | *P-Value* |
| Age, mean (SD), y | 58.05(10.58) | 56.85(13.02) | 0.1131 | 0.233 |  | 57.99(10.41) | 58.37(11.95) | 0.0333 | 0.707 |
| Female | 85(48.85) | 1821(48.07) | 0.0156 | 0.841 |  | 83(50.30) | 335(52.83) | 0.0586 | 0.561 |
| Body Mass Index, mean (SD), kg/m^2^ | 23.09(2.89) | 23.12(3.35) | 0.0108 | 0.904 |  | 23.11(2.93) | 23.15(3.40) | 0.0210 | 0.869 |
| ASA Class 3 or higher | 57(32.76) | 1098(28.99) | 0.0804 | 0.284 |  | 54(32.73) | 214(33.75) | 0.0484 | 0.804 |
| Preoperative Hemoglobin, mean (SD) | 137.30(15.56) | 134.14(15.73) | 0.2035 | 0.009 |  | 136.84(15.56) | 136.33(14.66) | 0.0042 | 0.709 |
| Preoperative Albumin, mean (SD) | 44.69(4.12) | 43.93(4.00) | 0.1835 | 0.015 |  | 44.57（4.14） | 44.53（3.82） | 0.0032 | 0.920 |
| Preoperative steroid use | 0(0) | 5(0.13) | 0.0372 | 0.632 |  | 0(0) | 0(0) | 0.0000 | 1.000 |
| Smoker | 29(16.67) | 791(20.88) | 0.1131 | 0.18 |  | 29(17.58) | 95(14.98) | 0.0786 | 0.413 |
| Drinker | 20(11.49) | 451(11.91) | 0.0129 | 0.87 |  | 20(12.12) | 72(11.36) | 0.0380 | 0.784 |
| Hypertension | 42(24.14) | 857(22.62) | 0.0354 | 0.641 |  | 41(24.85) | 160(25.24) | 0.0090 | 0.918 |
| Diabetes | 9(5.17) | 231(6.10) | 0.0418 | 0.617 |  | 9(5.45) | 48(7.57) | 0.0932 | 0.441 |
| COPD | 4(2.30) | 117(3.09) | 0.0527 | 0.554 |  | 4(2.42) | 16(2.52) | 0.0000 | 0.942 |
| Emphysema | 7(4.02) | 192(5.07) | 0.0532 | 0.537 |  | 7(4.24) | 23(3.63) | 0.0386 | 0.711 |
| Preoperative Pulmonary Inflammation | 30(17.24) | 728(19.22) | 0.0523 | 0.517 |  | 30(18.18) | 100(15.77) | 0.0655 | 0.455 |
| Pulmonary Arterial Hypertension | 7(4.02) | 145(3.83) | 0.0099 | 0.896 |  | 7(4.24) | 26(4.10) | 0.0154 | 0.935 |
| Other Pulmonary Disease | 6(3.45) | 103(2.72) | 0.04 | 0.565 |  | 5(3.03) | 22(3.47) | 0.0166 | 0.781 |
| Fluid Balance | 1505.75(433.39) | 1489.57(480.36) | 0.0373 | 0.663 |  | 1490.91(429.38) | 1491.32(459.59) | 0.0291 | 0.992 |
| Surgical Duration | 135.05(54.87)) | 145.4(67.55) | 0.1886 | 0.047 |  | 136.47(55.43) | 137.08(59.56) | 0.0094 | 0.907 |
| Anesthesia Duration | 171.02(60.50) | 176.83(69.18) | 0.0961 | 0.276 |  | 170.38(60.73) | 170.43(61.81) | 0.0151 | 0.992 |
| Surgical Type |  |  |  | 0.003 |  |  |  |  | 0.880 |
| Wedge Resection | 16(9.20) | 725(19.14) | 0.3432 |  |  | 16(9.70) | 71(11.20) | 0.0367 |  |
| Segmentectomy | 22(12.64) | 277(7.31) | 0.1604 |  |  | 19(11.52) | 62(9.78) | 0.0258 |  |
| Lobectomy | 133(76.44) | 2720(71.81) | 0.1091 |  |  | 127(76.97) | 493(77.76) | 0.0012 |  |
| Sleeve resection | 1(0.57) | 19(0.5) | 0.0097 |  |  | 1(0.61) | 3(0.47) | 0.0200 |  |
| Bilobectomy | 1(0.57) | 6(0.16) | 0.0551 |  |  | 1(0.61) | 1(0.16) | 0.0000 |  |
| Pneumonectomy | 1(0.57) | 41(1.08) | 0.0672 |  |  | 1(0.61) | 4(0.63) | 0.0000 |  |
| Surgical Approach |  |  | 0.1157 | 0.191 |  |  |  | 0.0454 | 0.658 |
| Thoracotomy | 16(9.20) | 475(12.54) |  |  |  | 15(9.09) | 65(10.25) |  |  |
| Video-assisted Thoracoscopic Surgery | 158(90.80) | 3313(87.46) |  |  |  | 150(90.91) | 569(89.75) |  |  |
| Use of Blood Product | 0(0) | 7(0.18) | 0.044 | 0.57 |  | 0(0) | 0(0) | 0.0000 | 1.000 |
| Use of Muscle Relaxants | 33(18.97) | 591(15.60) | 0.0858 | 0.234 |  | 30(18.18) | 114(17.98) | 0.0077 | 0.952 |

Abbreviations: GA, general anesthesia; GA+EA, general anesthesia combined with epidural anesthesia; ASD, absolute standardized difference; BMI, body mass index (calculated as weight in kilograms divided by height in meters squared); ASA, American Society of Anesthesiologists Physical Status classification; COPD, chronic obstructive pulmonary disease; SD, Standard deviation.

† *ASD, also referred to as ASMD (absolute standardized mean difference). Values < 0.1 indicate acceptable balance.

**Table S4. Secondary Outcomes in Patients Who received GA vs GA+EA in the Propensity Score Matched Cohort.**

|  | *GA+EA (n=165)* | *GA (n=634)* |  |  |
| --- | --- | --- | --- | --- |
| Postoperative Outcome | No.(%) | No.(%) | *P-*value^‡^ | adjusted *P-valu*e^§^ |
| **MACE** | *6（3.64）* | *37（5.84）* | *0.245* | *0.717* |
| New Cardiac Arrhythmia | *5（3.03）* | *26（4.10）* | *0.507* | *0.789* |
| Acute Myocardial Infarction | *2（1.21）* | *11（1.74）* | *0.64* | *0.815* |
| Congestive Heart Failure. | *0（0）* | *10(1.58)* | *0.933* | *0.972* |
| Others^a^ | *0（0）* | *11(1.74)* | *0.835* | *0.972* |
| **Hypotension** | *55(18.18)* | *26(2.52)* | *0.005* | *0.035* |
| **Slow Heart Rate** | *1(0.61)* | *7(1.10)* | *0.256* | *0.717* |
| **Pain** | *68(41.21)* | *404(63.72)* | *<0.001* | *<0.001* |
| **Opioid consumption**  **24MME**  **48MME**  **Total MME** | *24.9(19.7-30.3)*  *40(35-60)*  *75(75-100)* | *61.9(55.3-67.5)*  *121.9(109.8-131.4)*  *182.5(161.1-199.4)* | *<0.001* | *<0.001* |
| **ARI** | *3(1.82)* | *9(1.42)* | *0.438* | *0.789* |
| **Unplanned Tracheal Ontubation** | *1(0.61)* | *3(0.47)* | *0.323* | *0.754* |
| **Unexpected ICU Admission** | *0(0)* | *3(0.47)* | *0.972* | *0.972* |
| **Automatic Discharge** | *0(0)* | *1(0.16)* | *0.61* | *0.815* |
| **Hospitalization Costs，median(IQR)** | 42637.9(36385.5-48594.2) | 41345.4(34240.8-47422.4) | *0.132* | *0.616* |
| **Postoperative Hospital Stay, median (IQR)** | 4.8(3.7-6.7) | 4.8(3.7-6.6) | *0.467* | *0.789* |

Abbreviations: GA, general anesthesia; RA, regional anesthesia; MACE, major adverse cardiac events; ARI, acute renal injury; ICU, intensive care unit; IQR, interquartile range.

‡ *P-values were adjusted for multiple testing using the Bonferroni method.

§ P-value for continuous outcomes (costs, length of stay) derived from Wilcoxon rank-sum test.

**Table S5: Adjusted Analysis of Primary Outcomes by Anesthesia Type.**

| Primary Outcomes | Group | N(%) | HR (95% CI) | P-value |
| --- | --- | --- | --- | --- |
| PPCs | GA | 3788 | 1.00 |  |
|  | GA+RA | 5420 | 0.93(0.86-1.01) | 0.078 |
|  | GA+PRA  GA+PVB  GA+SAPB  GA+ICNB  GA+Other/combined techniques | 5246(100%)  281(5.36%)  1982(37.78%)  2118(40.37%)  865(16.49%) | 0.95(0.879-1.03)  0.77(0.61-0.98)  0.81(0.73-0.91)  0.92 (0.83-1.01)  1.06(0.94-1.20) | 0.221  0.03  <0.001  0.086  0.362 |
|  | GA+EA | 174 | 0.85(0.62-1.15) | 0.25 |

Abbreviations: GA, general anesthesia; RA, regional anesthesia; GA+PRA, general anesthesia combined with peripheral regional anesthesia; GA+PVB, eneral anesthesia combined with paravertebral block; GA+SAPB, general anesthesia combined with serratus anterior plane block; GA+ICNB, general anesthesia combined with intercostal nerve block; GA+EA, general anesthesia combined with epidural anesthesia; PPCs, postoperative pulmonary complications; HR, hazard ratio.

**Table S6: Results of the Sensitivity Analysis**

| Analysis | PPCs Definition | HR (95% CI) | P-value |
| --- | --- | --- | --- |
| Primary Analysis | Including "suspected pulmonary infection" | 0.93 (0.86-1.01) | 0.078 |
| Sensitivity Analysis | Excluding "suspected pulmonary infection" | 0.97 (0.89-1.05) | 0.48 |

Abbreviations: PPCs, postoperative pulmonary complications; HR, hazard ratio.

|  | GA+PVB vs GA | |  | GA+SAPB vs GA | |  | GA+ICNB vs GA | |  | GA+Others vs GA | |
| --- | --- | --- | --- | --- | --- | --- | --- | --- | --- | --- | --- |
|  | Before Matching | After Matching |  | Before Matching | After Matching |  | Before Matching | After Matching |  | Before Matching | After Matching |
| Characteristic | ASD^†^ | ASD^†^ |  | ASD^†^ | ASD^†^ |  | ASD^†^ | ASD^†^ |  | ASD^†^ | ASD^†^ |
| Age | 0.1775 | 0.0054 |  | 0.0947 | 0.0297 |  | 0.0216 | 0.0277 |  | 0.0538 | 0.0021 |
| Sex (Female) | 0.1 | 0.015 |  | 0.084 | 0.0065 |  | 0.0944 | 0.0185 |  | 0.0258 | 0.0062 |
| BMI | 0.0311 | 0.0056 |  | 0.0309 | 0.0009 |  | 0.0433 | 0.0069 |  | 0.0540 | 0.0077 |
| ASA Class 3 or higher | 0.095 | 0.024 |  | 0.1476 | 0.0259 |  | 0.1210 | 0.0082 |  | 0.0816 | 0.022 |
| Preoperative Hemoglobin | 0.0866 | 0.0284 |  | 0.1235 | 0.0124 |  | 0.1467 | 0.0195 |  | 0.0288 | 0.014 |
| Preoperative Albumin | 0.105 | 0.0076 |  | 0.2150 | 0.0044 |  | 0.2653 | 0.0196 |  | 0.0358 | 0.0063 |
| Preoperative steroid use | 0.0377 | 0.0000 |  | 0.0098 | 0.034 |  | 0.0026 | 0.0129 |  | 0.0207 | 0.0161 |
| Smoker | 0.024 | 0.012 |  | 0.08 | 0.0438 |  | 0.0472 | 0.0247 |  | 0.0151 | 0.0026 |
| Drinker | 0.120 | 0.044 |  | 0.019 | 0.0290 |  | 0.0027 | 0.0284 |  | 0.0106 | 0.0103 |
| COPD | 0.007 | 0.0255 |  | 0.0504 | 0.0082 |  | 0.0043 | 0.0028 |  | 0.0609 | 0.0119 |
| Emphysema | 0.04 | 0.037 |  | 0.0727 | 0.0128 |  | 0.0035 | 0.0154 |  | 0.0099 | 0.0099 |
| Preoperative Pulmonary Inflammation | 0.018 | 0.0008 |  | 0.0103 | 0.0245 |  | 0.0371 | 0.0076 |  | 0.0066 | 0.0071 |
| Pulmonary Arterial Hypertension | 0.0145 | 0.0113 |  | 0.0641 | 0.000 |  | 0.0725 | 0.0149 |  | 0.0481 | 0.01 |
| Other Pulmonary Diseases | 0.015 | 0.0289 |  | 0.107 | 0.000 |  | 0.0990 | 0.0199 |  | 0.0543 | 0.0014 |
| Fluid Balance | 0.0968 | 0.0856 |  | 0.1718 | 0.0027 |  | 0.1363 | 0.0181 |  | 0.0304 | 0.0032 |
| Surgical Duration | 0.089 | 0.0473 |  | 0.4565 | 0.0237 |  | 0.2475 | 0.02 |  | 0.0381 | 0.0067 |
| Anesthesia Duration | 0.0924 | 0.0489 |  | 0.5008 | 0.0205 |  | 0.2786 | 0.026 |  | 0.0185 | 0.0106 |
| Surgical Type |  |  |  |  |  |  |  |  |  |  |  |
| Wedge Resection | 0.025 | 0.035 |  | 0.0556 | 0.0066 |  | 0.1006 | 0.0057 |  | 0.0695 | 0.001 |
| Segmentectomy | 0.067 | 0.0052 |  | 0.0863 | 0.0217 |  | 0.0670 | 0.0017 |  | 0.0913 | 0.0493 |
| Lobectomy | 0.0294 | 0.0269 |  | 0.0771 | 0.0081 |  | 0.1042 | 0.0103 |  | 0.0100 | 0.0266 |
| Sleeve resection | 0.055 | 0.0029 |  | 0.0901 | 0.0139 |  | 0.0298 | 0.000 |  | 0.0263 | 0.0016 |
| Bilobectomy | 0.0413 | 0.0000 |  | 0.0481 | 0.0241 |  | 0.0512 | 0.0447 |  | 0.0441 | 0.0000 |
| Pneumonectomy | 0.0014 | 0.0000 |  | 0.1419 | 0.0000 |  | 0.3216 | 0.0316 |  | 0.0305 | 0.0292 |
| Surgical Approach | 0.014 | 0.014 |  | 0.7235 | 0.03 |  | 0.6252 | 0.0031 |  | 0.3053 | 0.0123 |
| Use of Blood Product | 0.0446 | 0.0000 |  | 0.0038 | 0.0000 |  | 0.0185 | 0.0274 |  | 0.0276 | 0.0049 |
| Use of Muscle Relaxants | 0.0661 | 0.0311 |  | 0.0917 | 0.0016 |  | 0.0982 | 0.0044 |  | 0.0096 | 0.0066 |

**Table S7. Baseline Covariate Balance (ASD) Before and After Propensity Score Matching (GA vs other subgroups)**.

Abbreviations: GA, general anesthesia; GA+PVB, eneral anesthesia combined with paravertebral block; GA+SAPB, general anesthesia combined with serratus anterior plane block; GA+ICNB, general anesthesia combined with intercostal nerve block; ASD, absolute standardized difference; BMI, body mass index (calculated as weight in kilograms divided by height in meters squared); ASA, American Society of Anesthesiologists Physical Status classification; COPD, chronic obstructive pulmonary disease

† *ASD, also referred to as ASMD (absolute standardized mean difference). Values < 0.1 indicate acceptable balance.
